# Study on detonation characteristics of pulverized coal and evolution law of detonation residue

**DOI:** 10.1038/s41598-024-62489-y

**Published:** 2024-05-22

**Authors:** Jing Guo, Shirong Ge, Yinan Guo, Jiayu Liang, Ruibo Yang

**Affiliations:** School of Energy and Mining, China University of Mining and Technology (Beijing), Beijing, 100083 China

**Keywords:** Coal, Detonation residue, XRD, FTIR, Rotational detonation mechanism, Coal, Chemical engineering

## Abstract

This study explores the detonation characteristics and compositional changes of pulverized coal, focusing on its use in Rotary Detonation Wave (RDW) technologies. While pulverized coal has shown high fuel efficiency in RDW settings, transitioning from theory to practical detonation engineering presents substantial scientific and technical hurdles. A key issue is the reprocessing of detonation byproducts for in-situ coal mine gob filling, a topic that has received little attention. Utilizing advanced methods like X-ray diffraction (XRD) and Fourier transform infrared spectroscopy (FTIR), this paper investigates the micro-morphology, composition, and aromatic structures of gas–solid products pre and post-detonation at the Tashan Coal Mine's 2305 working face. Results indicate that coal dust from the underground mining face has enhanced detonation characteristics, with the addition of coal powder fuel extending the gas detonation limits. This benefits economic aspects by reducing reliance on gas fuel and lowering detonation fuel costs. The highest recorded detonation wave velocity was 2450 m/s, 14.8% greater than that of coal dust from external sources, suggesting more effective energy release and pressure gain. Furthermore, the study links detonation combustion intensity to coal's aromatic properties, noting a post-detonation aromaticity index (*I*) of 0.4941. This indicates an improvement in the aromatic structure under high-temperature conditions, vital for coal's reactivity and energy efficiency in RDW applications. This research not only deepens the understanding of coal dust combustion mechanisms but also advances clean coal utilization and deep coal fluidization mining, addressing significant RDW technological challenges.

## Introduction

Nowadays, the detonation combustion cycle (Zel’dovich) has garnered increasing attention from scientists due to its superiority over the constant pressure cycles (Brayton/Humphrey)^1–3^. Detonation combustion is characterized by its rapid detonation velocity, high pressure gain, and efficient energy release^4–6^. Notably, it simplifies the internal structure of detonation chambers and eliminates unnecessary mechanical rotating parts, sparking widespread research interest^7–11^. To achieve better energy conversion performance, scientists have explored various technologies to improve detonation combustion chambers. Among these, the Pulse Detonation combustion (PDC) device is relatively mature, featuring a simple structure, high operating frequency, and reliable performance. However, the need for multiple ignitions is not conducive to multi-stage utilization. Oblique Detonation combustion (ODC) remains in the theoretical research phase and is primarily applied in aerospace propulsion engineering. Besides these two types, the rotating detonation combustion (RDC) is also gaining attention for its ability to maintain a stable continuous detonation state after a single ignition, making it one of the most promising energy conversion devices available today^12–16^.

**Figure 1:**
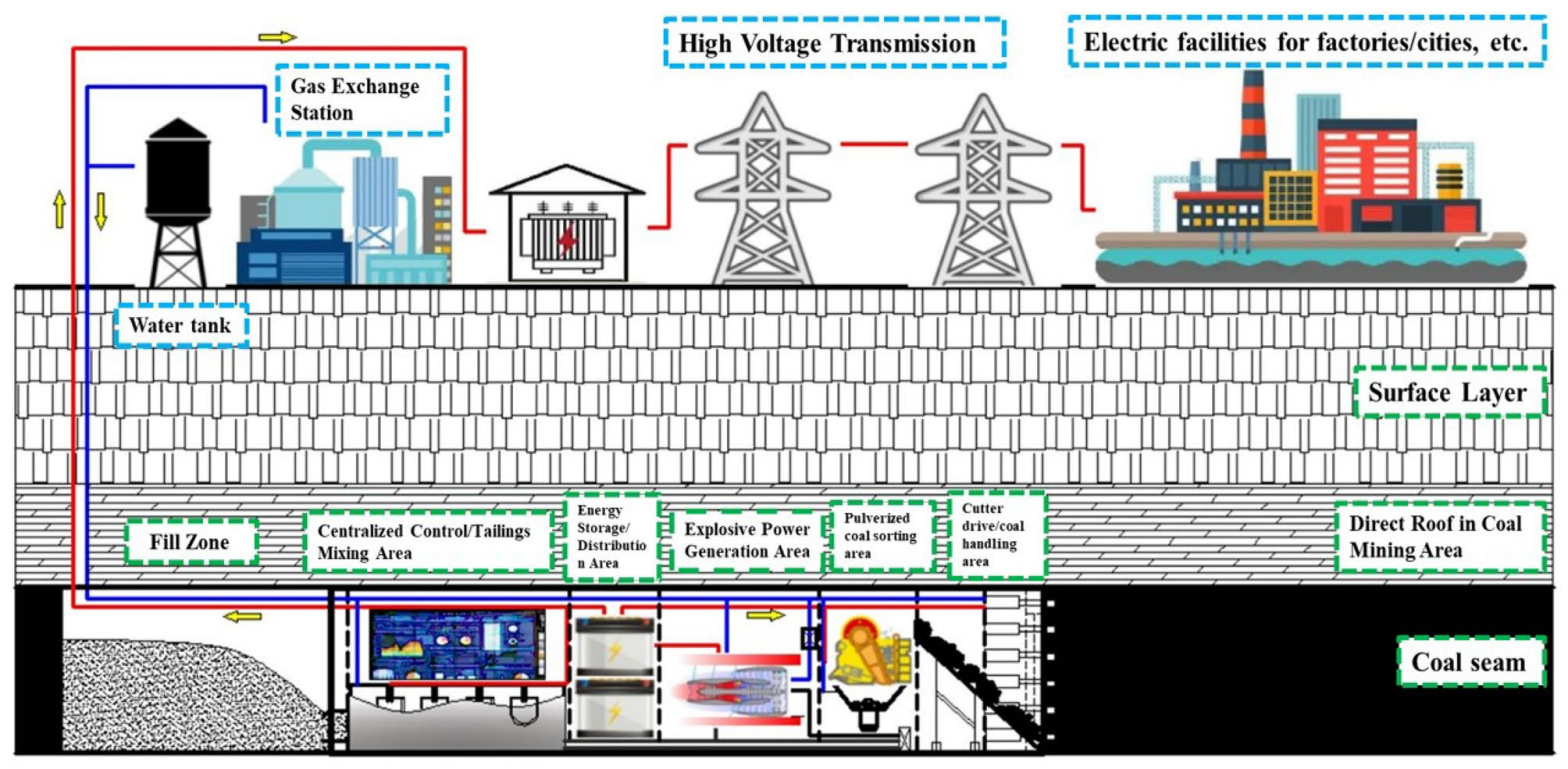
Concept of fluidized mining of deep coal resources.

Introduced as early as 2016, the concept of in-situ fluidized mining of deep coal integrates various modules including mining, support, coal sorting, fluidization transformation, and energy storage into a singular conceptual mining apparatus^17–21^. The cornerstone of this innovative extraction method is the detonation combustion of pulverized coal, aiming to transmute deep-earth coal resources into alternative energy forms, such as the conversion of fossil fuels into electrical energy, as depicted in Fig. 1. The primary objective is to harness the RDC device's capacity for efficiently liberating energy from pulverized coal situated deep underground, thereby revolutionizing the approach to coal energy utilization^22,23^. Due to its significant characteristics, which include exceptionally high thermal cycle efficiency, an extraordinarily rapid heat release rate, and notably efficient pressure gain, detonation combustion stands poised to revolutionize the field of fluidized bed mining for deep coal resources. This technology holds the potential to significantly enhance the efficiency and effectiveness of extracting deep coal resources, positioning it as a highly innovative solution for energy acquisition in challenging environments. The unique properties of detonation combustion could transform it into a key technological advancement, making it a pivotal option in the quest for more efficient and sustainable energy production methods in the mining sector.

The concept of rotating detonation was first discovered by Voitsekhovskii from the former Soviet Academy of Sciences in 1959 while studying transverse detonation waves^24^. He ignited a mixture of argon-diluted C_2_H_2_/O_2_ in a disc-shaped combustion chamber and successfully captured the structure of the rotating detonation wave inside the chamber using a fully compensated schlieren photography technique. Since 2000, advancements in fundamental disciplines such as fluid dynamics and materials mechanics have led to a significant surge in research outcomes related to Rotating Detonation Engines (RDEs). As a pioneer in the research of Rotating Detonation Engines (RDE), Russia has amassed a significant body of knowledge. The Frolov team from the Russian Academy of Sciences has conducted extensive experimental research using a pulse wind tunnel on hydrogen-air mixtures for RDEs. Their studies have confirmed the feasibility of achieving stable detonation waves in scramjet engines operating at Mach numbers between 4 and 8, documenting detonation wave speeds of up to 1200 m per second and a maximum thrust of 2200 newtons^25,26^. GHKN Corporation and Locke-Dyne Inc. researched RDRE using methane, ethane, and ethylene with oxygen. They tested various flow rates and fuel ratios. High-speed photos showed 5–8 detonation waves in the chamber, reaching 60–72% of the theoretical C–J speed. Studies indicate RDRE's specific impulse might achieve 68–85% of an ideal rocket engine's performance at sea level^27^. It is evident that the primary fuels used in Rotating Detonation Engines (RDEs) are gaseous and liquid forms, predominantly applied in military and aerospace propulsion technologies.

Compared with the study of detonation as propulsion technology, the research and development of in-situ detonation combustion of pulverized coal started relatively late, and its research is still in the experimental and simulation stage. Zhu et al.^28^ simulated the two-phase flow field in a Rotating Detonation Engine (RDC) using carbon/H_2_ as fuel, revealing that a low-temperature methane gap replaces deflagration, enhancing the maximum Rotating Detonation Wave (RDW) to achieve a wave velocity performance of 1780 m/s. Salvadori et al.^29^ used the Euler-Lagrangian dense particle formula to simulate the discrete phase in detonation combustion. The results indicate that the detonation wave mainly relies on the heat released by fuel detonation for propagation. Li et al.^30^ studied the equivalence ratio of methane-solid two-phase fuel using CFD fluid software (ANYS-FLUENT), and found that the equivalence ratio affects the stable propagation of RDW. Pioneers like Bykovskii et al.^31,32^ and Dunn et al.^32^ have successfully operated rotating detonation engines (RDC) using coal/H_2_ as the primary solid fuel during the experimental phase. Bykovskii et al.^31^ designed RDC of varying physical sizes and explored the detonation characteristics of coals with different qualities (composition, particle size) using H_2_ as an auxiliary burst methane. Dunn et al.^32^ confirmed that coal dust enhances detonation in RDC and that the detonation heat generated by the coal dust detonation is linearly related to the amount of coal dust added. Xu et al.^33^ conducted experimental studies on anthracite/H_2_/oxygen fuel in RDC, revealing that volatile coal dust particles can increase the operating range of the rotating detonation wave (RDW) by at least 15%. Anthracite dust improved the velocity and peak pressure of the RDW, and an increase in equivalence ratio initially increased and then decreased the thrust of the RDC, demonstrating the existence of an optimal fuel equivalence ratio. Extensive numerical simulations and experimental research have confirmed the feasibility of using pulverized coal in RDW applications, demonstrating high fuel efficiency^30–33^.

Comprehensive numerical simulations and experimental research underline the viability of utilizing pulverized coal in Rotary Detonation Wave (RDW) applications, showcasing notable fuel efficiency. While this technology has transcended the theoretical research phase in numerous countries, formidable scientific challenges and pivotal technological advancements remain imperative for its transition to practical engineering applications. An urgent technical challenge involves the reprocessing of the byproducts of pulverized coal detonation combustion to serve as raw materials for in-situ filling of coal mine gob. Current research on the products of pulverized coal post-RDC detonation remains scant. Shi et al.^35^ employed the Euler–Lagrange method to examine the dynamic detonation of rare coal coke particles, revealing that coal powder particles smaller than 20 μm, owing to their porosity, facilitate the formation of pressure waves, thereby influencing the shock wave state. Lin et al.^36^ investigated the explosion characteristics of pulverized coal using a spherical explosion device, with infrared spectroscopy analysis of the explosion residue highlighting the pivotal role of fixed carbon components in the explosion process. Li et al.^37^ explored the residues generated by coal dust during detonation, and found significant changes in the content of aromatic –CH and C=C compared to the original coal dust. The research emphasizes the important impact of aliphatic C–H and oxygen-containing functional groups on the detonation intensity of coal dust, while also indicating the presence of combustible components in the residues, which may trigger secondary detonation. Deflagration and detonation fundamentally differ in their combustion processes, leading to theoretically disparate outcomes in their reaction products. Currently, research specifically analyzing the products of coal dust detonations remains scant.

In summary, systematic studies on the intensity of coal dust detonations and the compositional changes in their residues remain scarce. Further research is essential to elucidate the mechanisms of energy release during coal dust detonations, addressing this critical issue. To address this issue, this paper utilizes coal from the 2305 working face of Tashan Coal Mine as a raw material, analyzing the produced raw coal. Employing X-ray diffraction (XRD), Fourier transform infrared spectroscopy (FTIR), among other characterization techniques, this study compares the micro-morphology, composition, and aromatic structure of gas–solid products before and after detonation. Additionally, it discusses the evolutionary trajectory and laws governing the coal from the 2305 working face of Tashan Coal Mine during detonation. This investigation not only provides data support for deep coal fluidization mining and the clean utilization of coal but also pioneers new avenues for research.

## Experiments and methods

### Materials

**Figure 2:**
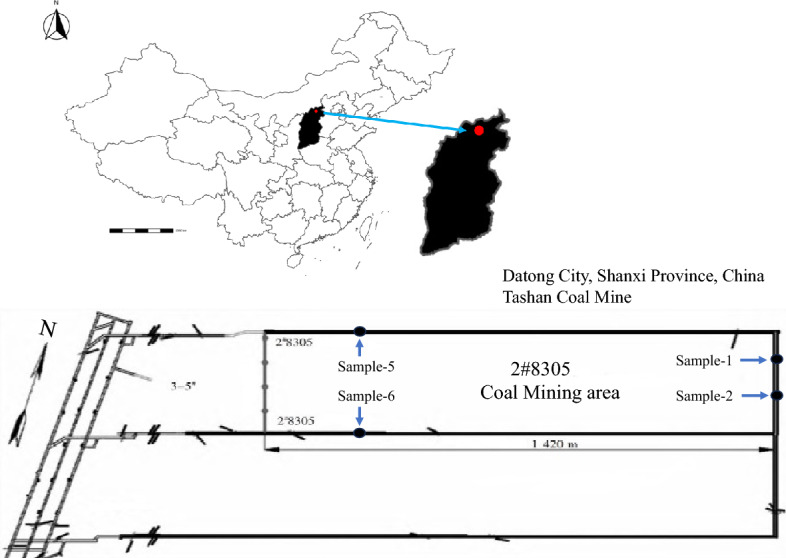
Coal sample sampling location and physical characteristics.

In this study, the pulverized coal particles of non-caking coal are selected. The coal sample is provided by the Tashan coal mine, Datong City, Shanxi Province, China (as shown in Fig. 2). The coal sample was dried and ground. In compliance with standard GB/T-30732-2014, the characteristic parameters of the four types of coal samples were industrially analyzed, and the results are presented in Table 1. Four types of raw coal are designated as Sample1, 2, 5, and 6, with the corresponding exploded coal samples named Sample3, 4, 7, and 8, respectively. At least five duplicate tests areconducted. The experiment utilized methane with a purity of 99.995% as the methane source.

**Table 1:** Results of industrial analysis for coal samples (air dried basis).

Sample	Proximate analysis (%)				Elemental analysis (%)					H/C
	M_ad_	A_ad_	V_ad_	FC_ad_	C	H	O	N	S	
1	1.23	34.51	3.78	60.48	85.49	4.35	16.89	0.95	0.21	0.72
2	1.54	34.16	5.64	58.66	78.43	4.28	18.43	1.05	0.35	0.74
5	1.80	8.16	2.34	67.70	76.45	3.61	15.72	1.52	0.68	0.65
6	1.65	28.25	6.42	63.68	81.65	4.55	12.95	1.67	0.35	0.70

### Experimental equipment and methods

**Figure 3:**
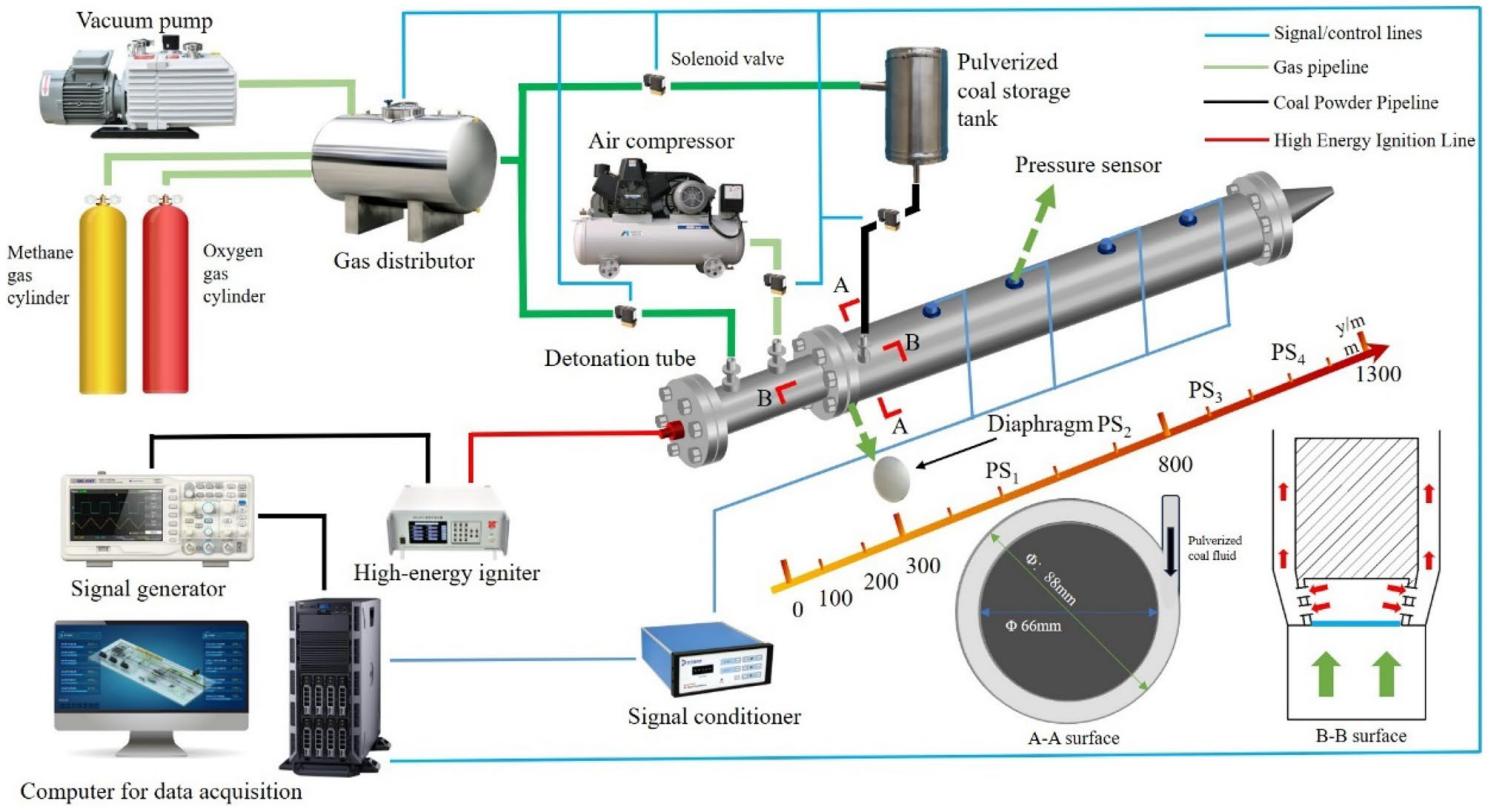
Experimental system for the powder coal/CH_4_/O_2_ rotating detonation engine (RDC).

Experimental equipment and methods Fig. 3 depicts a schematic of the experimental setup, which was constructed and operated in compliance with the safety standards ISO 17025 and ASTM E456. The setup comprises a custom-built RDC main detonation chamber, fuel supply system, oxidizer supply system, ignition system, and control and data acquisition system. Each component of the system was calibrated and validated following the procedures outlined in ISO 5167, ensuring the accuracy and reliability of our experimental data. The RDC combustors are comprised of a fuel chamber, methane chamber inlet, and powder inlet. Figure 2 shows a sectional view of the RDC combustor, which has a coaxial ring structure with an outer ring diameter of 88 mm, an inner ring diameter of 66 mm, and a combustor length of 1000 mm. During operation, fuel and oxygen are pre-injected into the RDC combustor and then enter the RDC through evenly distributed orifices, where they participate in mixing and detonation. The coal powder supply system, as shown in Fig. 3, mainly consists of methane pipelines, powder storage chambers, piston components, and stepper motor. The fuel supply process is as follows: firstly, ultrafine coal powder is injected into the storage chamber, then the coal powder is compacted by the stepper motor and piston components. Methane is introduced into the storage chamber at a set flow rate, carrying the coal powder into the RDC detonation chamber to complete one fuel injection. Both the oxygen pipeline to the RDC and the hydrogen pipeline to the fluidized bed are equipped with sonic nozzles to achieve stable methane flow rates. In the experiment, the upstream pressure of each pipeline sonic nozzle is adjusted. In addition, Endress Hauser mass flow meters (Pro-mass F300) are used to measure the mass flow rates of oxygen and hydrogen. The maximum error of the mass flow meter is ± 0.2%. During the RDC operation, 4 high-frequency pressure sensors (PS_1–4_) installed on the detonation tube to monitor the continuous detonation state of lignite coal powder. The photodetector sensor used is of CKG100 type, with an excitation voltage of 4 V, a response time of less than 100us, and a spectral wavelength acquisition range of 150–980 nm. The pressure sensor used is of PCB-113B26 type, with a response time of less than 0.5 us, a sampling frequency of 500 kHz, a sensitivity of 0.7–1.5 V/MPa, and a maximum range of 6.8 MPa. To verify the quality flow of the fuel supply system, a series of fluidization experiments are conducted in advance to calibrate the relationship between the methane source flow and the coal powder mass flow.

In the study, consistent conditions were maintained during the detonation process, except for the particle size and type of coal powder. The oxidizer and detonation-supporting gas (O_2_/CH_4_) were set at the highest equivalence ratio to achieve the strongest detonation effect^34^. X-ray diffraction analysis of raw and pulverized coal after detonation was performed using the Rigaku Ulima 5 X-ray powder diffractometer from Japan. The equipment's diffraction Angle 2θ ranged from 10° to 80°, and the mineral powder needed to be finer than 200 meshes. Data were analyzed by Jade6.0 to identify the main mineral types in the pulverized coal. Active groups in coal samples were determined using the Fourier transform infrared spectrometer TENSOR-27 from Germany. Pulverized coal under 200 mesh was mixed and ground with KBr at a ratio of 1:200. The spectrum was measured in the range of 400–4000 cm^−1^, and the collected FTIR data were recorded.

### Experimental procedure

**Figure 4:**
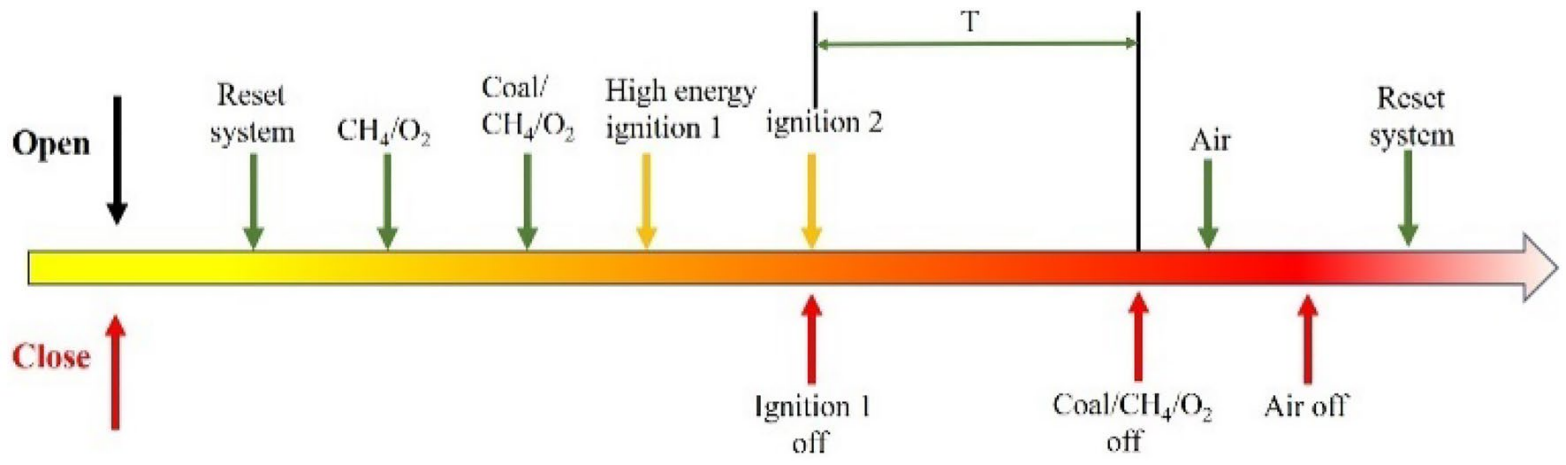
Lignite powder /CH_4_/O_2_ rotary detonation engine (RDC) operating time sequence.

Figure 4 shows the sequential operation time of the self-made RDC using coal/methane fuel. The arrows above the time axis indicate opening, while the arrows below the time axis indicate closing. During the experiment, the coordinated operation of the various parts of the experimental system is achieved by controlling the spark igniter, motor, and various solenoid valves through the control system. A complete engine operation process usually includes fuel pre-injection, ignition, RDC detonation chamber operation, and detonation chamber extinguishing. The supply of coal powder fuel requires the transportation of methane. Therefore, before supplying coal powder to the fluidized bed, the stable supply of methane should be ensured. The engine ignition process is completed by pre-blasting the methane/oxygen mixture through the pre-blast tube and igniting it with a spark plug. Subsequently, after a certain development time, the coal/methane/oxygen mixture will form a stable non-homogeneous detonation. To prevent the pressure sensor from being continuously damaged by high temperatures, the experimental duration of the detonation process is preset to Ata = 725 ms. At the end of the experiment, the fuel supply is first cut off, while the oxygen is supplied for a period of time to blow out the detonation and cool the RDC detonation chamber.

## Results and discussion

### Analysis of different coal dust detonation processes

A thorough analysis of the experimental data confirms the consistent propagation of coal dust/methane/oxygen detonation waves under the four experimental conditions shown in Table 2. Therefore, this paper selects a typical experimental result to analyze the detonation process of coal (sample 1).

**Table 2:** RDW high-frequency pressure signals collected by PS_1–4_.

Sample	*d*_002_/nm	*L*_c_/nm	*f*_a-XRD_	*N*_ave_
1	0.3517	1.834	0.571	5.125
2	0.3495	1.651	0.612	5.657
3	0.3436	0.975	0.684	4.951
4	0.3432	0.887	0.713	5.024
5	0.3554	1.323	0.553	5.366
6	0.3571	1.248	0.492	5.412
7	0.3412	0.654	0.748	4.859

**Figure 5:**
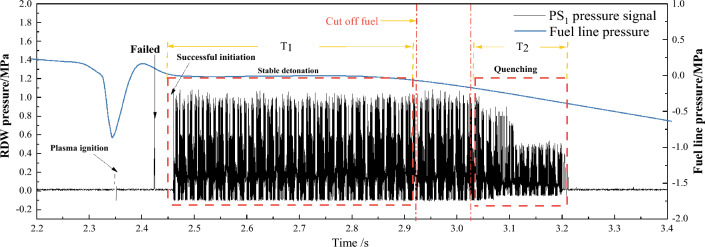
Fuel pipe pressure and RDW high frequency pressure data (sample 1).

Figure 5 presents the fuel line pressure within the RDC detonation chamber and the corresponding high-frequency pressure distribution for pulverized coal. The fuel line pressure reflects real-time data between the fuel storage room and the RDC detonation chamber. Pulverized coal, being more challenging to ignite compared to liquid and methane fuels, risks ignition failure due to inadequate fueling. In our experiments, a continuous rotating detonation wave (RDW) could be established after one or multiple detonations. The experimental process entails at least one ignition detonation, successfully transitioning to a continuous RDW through a pre-detonation phase. Given the non-premixed nature of the fuel supply, the heterogeneous provision of lignite coal powder can cause RDW pressure fluctuations. Moreover, an inverse correlation between the RDW's high-frequency pressure data and the fuel line orientation suggests that fuel supply characteristics significantly affect the RDW. In this study, after the onset of RDW, the duration of the stable propagation phase was set as T_1_, and the sudden extinguishment phase of RDW following fuel depletion in the pipelines and detonation chamber was designated as T_2_. The Rotating Detonation Wave (RDW) formed within 2.46 s during the experiment, with an initial duration of approximately 0.465 s until the fuel supply was cut off around 3.05 ms into the test. Despite the cutoff, the RDW continued to propagate steadily in the Rotating Detonation Engine (RDC) detonation chamber for about 100 ms due to the presence of unburned methane and solid fuels in the fuel supply chamber, pipelines, and detonation chamber. Subsequently, the detonation wave rapidly transitioned to deflagration until extinction, a process lasting approximately 200 ms (T_2_).

**Figure 6:**
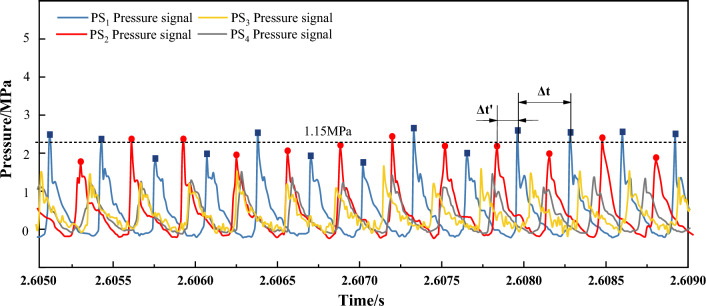
RDW high-frequency pressure signals collected by PS_1–4_.

Figure 6 illustrates the partial pressure curves measured by four sensors PS_1–4_ during the stable propagation phase of the RDW between 2.6050 and 2.6090 s. The pressure peak recorded by sensor PS_1_, positioned closest to the detonation point, is significantly higher compared to the progressively distant sensors PS_2_, PS_3_, and PS_4_. The time difference between adjacent pressure peaks at sensor P_1_ is denoted as Δt, while Δtʹ represents the time difference between the peak pressures at PS1 and PS2. With Δtʹ approximately half of Δt and considering the equal 200 mm distance from sensor PS1 to both the detonation point and sensor PS2, it can be inferred that the RDW propagation from detonation to sensors PS2 follows a linear acceleration process.

**Figure 7:**
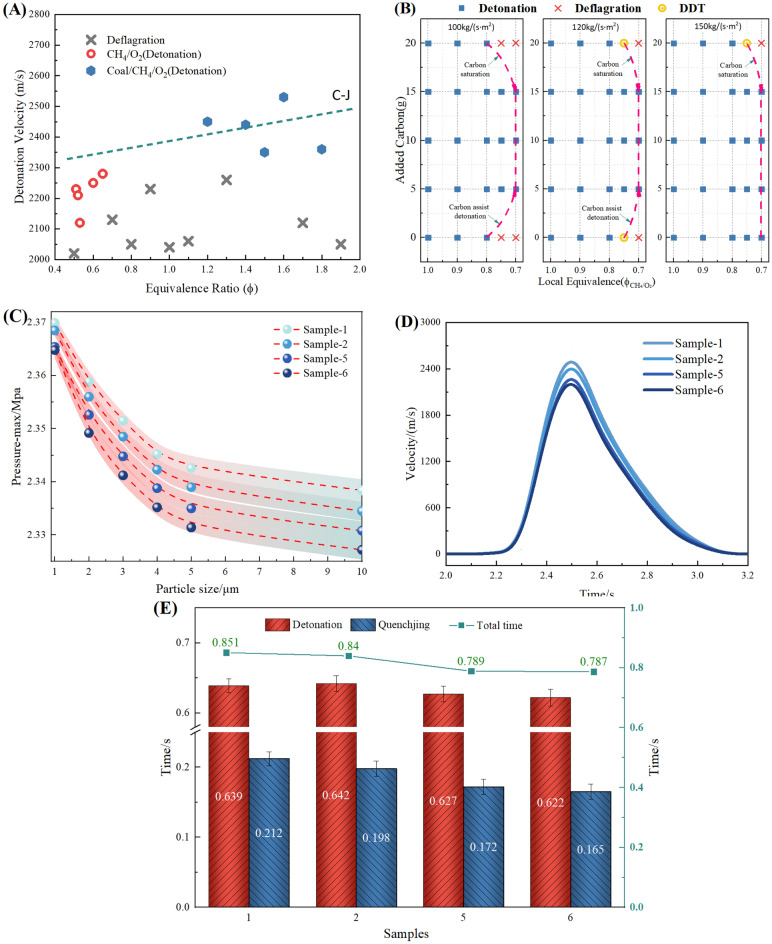
Detonation character istics of pulverized coal (**A**: Comparison of oxygen-methane-coal detonation velocity with theoretical calculated CJ velocity; **B**: Detonation state of carbon concentration and CH_4_/O_2_ equivalent ratio at different fuel flows; **C**: Relationship between coal particle size and maximum detonation pressure; **D**: Detonation wave propagation velocity; **E**: Detonation time of different coal samples).

Due to the difficulty of obtaining detonation burning states under experimental conditions, it is necessary to compare and reference the experimental data according to the classical detonation C-J theory. All C-J wave velocities were computed on NASA's Chemical Equilibrium and Applications thermodynamic calculator^38^. Figure 7A shows a comparison of the measured detonation velocity of the oxygen-methane-coal seed test with the calculated theoretical oxygen-methane-coal detonation velocity. When the detonation state is in “Deflagration”, it is considered that under this operating condition, detonation is not a self-sustaining detonation state, and at the same time, the detonation wave velocity is less than 60% of the theoretical value of the C–J detonation wave velocity. Figure 7B presents the detonation limits under three different gas–solid fuel flow conditions, namely 100 kg/(s m^2^), 120 kg/(s m^2^), and 150 kg/(s m^2^) operating conditions. It is important to note that the addition of coal powder fuel has increased the gas detonation limits, which is advantageous in reducing the dependence on gas fuel. This has economic benefits as it lowers the overall cost of detonation fuel consumption. Based on the data, as the amount of carbon powder added increases or the Φ_CH4/O2_ = 0.7 local equivalence ratio decreases, within the low flow range (100 kg/(s m^2^), 120 kg/(s m^2^)), there exist two types of detonation limits: carbon saturation and carbon-assisted detonation. Around Φ_CH4/O2_ = 0.7, the detonation sensitivity is significant. With the increase of fuel flow (150 kg/(s m^2^)), the addition of carbon powder effectively raises the detonation limit, and when Φ_CH4/O2_ = 0.75, only the carbon saturation detonation limit state exists. This phenomenon has previously been observed in a pulse detonation engine, where a purely thermodynamic approach revealed an optimal carbon addition concentration, as indicated by the carbon addition and a 0.7 CH_4_/O_2_ equivalence ratio.

The previous discussion focused on the entire process of detonation, and Fig. 7C–E specifically explored the detonation propagation characteristics of four coal samples. For solid fuel, particle size is a key factor affecting detonation. Figure 7C shows the relationship between different particle sizes and the maximum detonation pressure. According to the error confidence interval and data change, the change trend of detonation pressure of different particle sizes is basically consistent. For solid fuel, as the particle size decreases, P_max_ gradually increases. This indicates that under certain conditions, smaller particle sizes present a larger solid comparison area, which provides better contact conditions for solid fuel, detonation gas, and oxidants, thereby producing larger detonation characteristics. In this experimental setup, the maximum detonation pressure of the coal dust on the working face (Sample-1, 2) is at least 0.2% higher than that of the external coal dust on the working face (Sample-5, 6), with a particle size of 1 μm showing P_max_ of 2.373Mpa. Figure 7D shows the maximum propagation velocity of different coal dust detonations. It can be seen that the maximum detonation wave propagation velocity of the coal dust (Sample-1,2) inside the working face is at least 2450 m/s, which is 14.8% higher compared to the maximum velocity of the detonation wave of the coal dust (Sample-5, 6) outside the working face at 2135 m/s. Similarly, the coal dust inside the coal mining working face exhibits high detonation wave propagation characteristics.

During the detonation process, the duration of self-adaptive detonation combustion is a crucial parameter to explore the entire process. For the 4 types of coal powder, the experimental detonation process generally lasted for at least 0.787 s (as shown in Fig. 7E). The addition of the error bar reduces the error caused by multiple detonation experiments and enhances the credibility of the data change trend It can be observed that, with the supply of gas–solid fuel, a stable detonation process of at least 0.622 s can be generated. Similarly, the duration of detonation for internal coal powder (Sample-1, 2) is at least 2.73% higher than that of external coal powder (Sample-5, 6). Higher adaptive detonation time is crucial for the subsequent conversion of detonation energy, including improving prerequisites for mechanical and magnetohydrodynamic (MHD) power generation. Due to the complex physical and chemical changes involved in the process of coal dust detonation, it is necessary to conduct in-depth research based on its solid residue. At the mechanistic level, it is important to explore and explain the characteristics of the four types of coal dust detonation.

### Analysis of solid products of pulverized coal detonation

#### XRD analysis of solid products of pulverized coal detonation

XRD diffraction analysis enables the determination of the phase composition and microcrystalline structural parameters of raw coal from the same underground workface and different locations. This facilitates a deeper understanding of the fundamental structural characteristics of coal samples from various positions within the same coalfield, both pre- and post-detonation.

**Figure 8:**
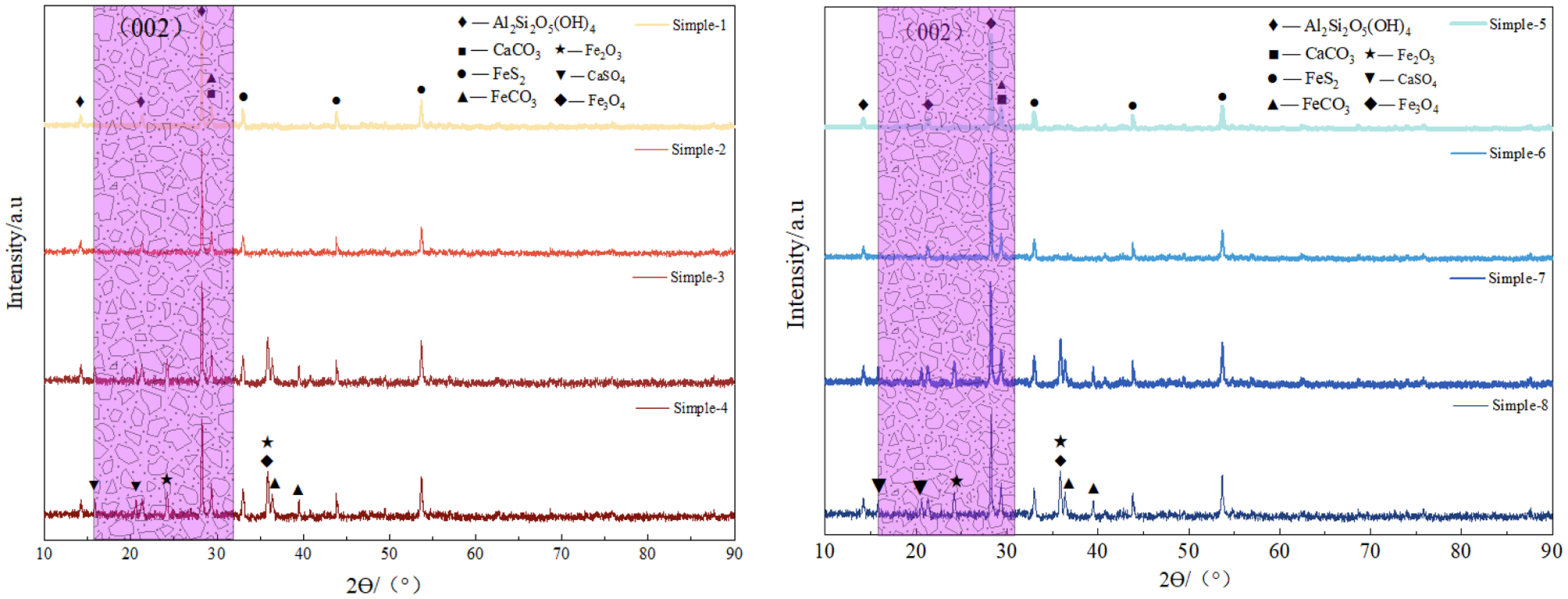
XRD analysis of detonation and non-detonation products from different coal samples.

The XRD spectra of the coal sample before and after the detonation are shown in Fig. 8. It can be observed that the main minerals present before the detonation are quartz (SiO_2_), kaolinite (Al_2_Si_2_O_5_(OH)_4_), pyrite (FeS_2_), and siderite (FeCO_3_). After the detonation, the diffraction peak intensity of kaolinite and siderite in the coal sample decreased or even disappeared, while the diffraction peak intensity of hematite increased. At the same time, andalusite (Al_2_SiO_5_) and gypsum (CaSO_4_) appeared. This is because kaolinite loses its hydroxyl groups and transforms into amorphous metakaolinite at high temperatures during the detonation, and clay minerals transform into andalusite (Al_2_SiO_5_), while high-temperature phase metal oxides such as magnetite (Fe_3_O_4_) and hematite (Fe_2_O_3_) are produced.

**Figure 9:**
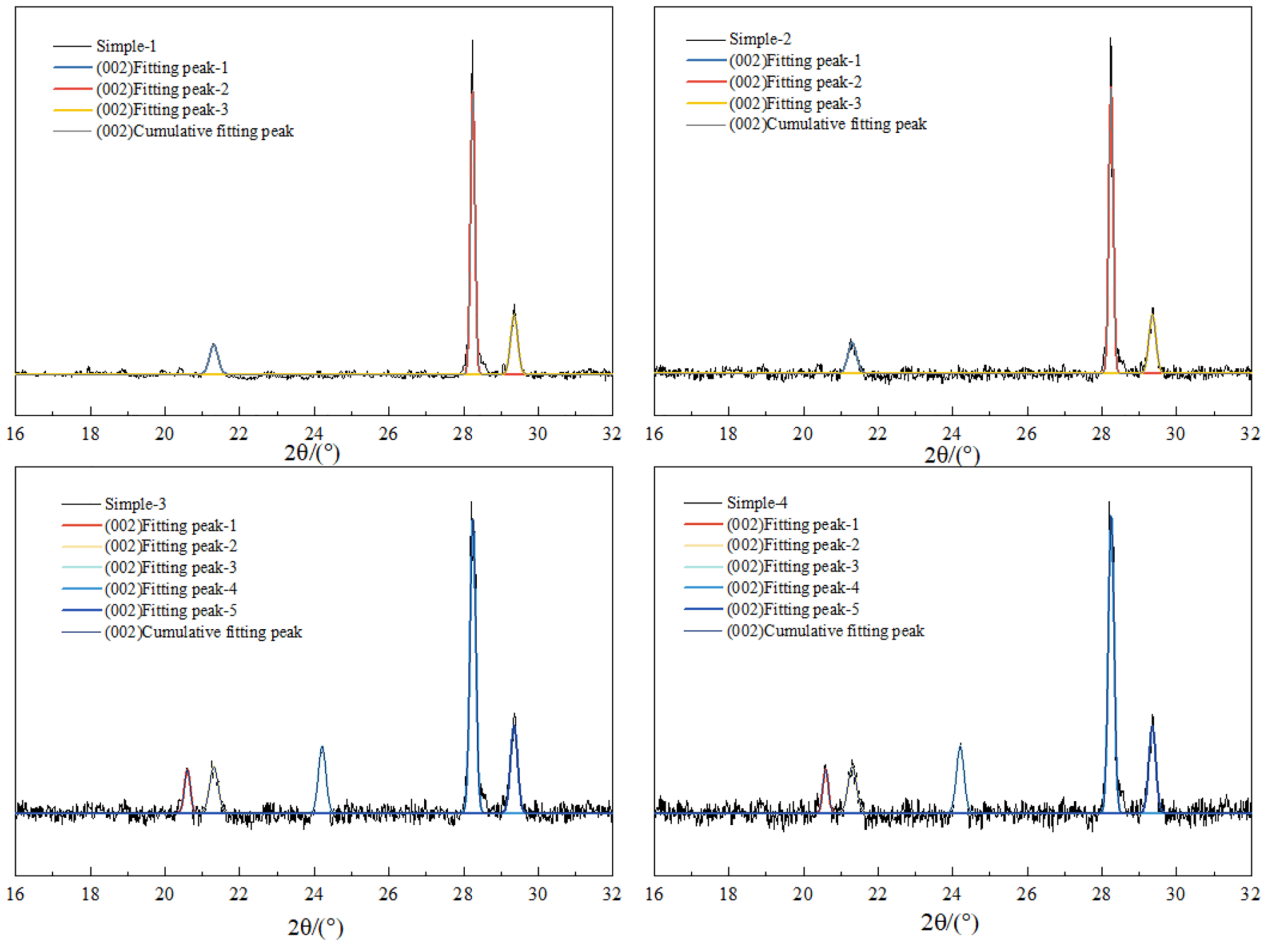
XRD curve fitting results for 002 peak of coal samples (1–4).

**Figure 10:**
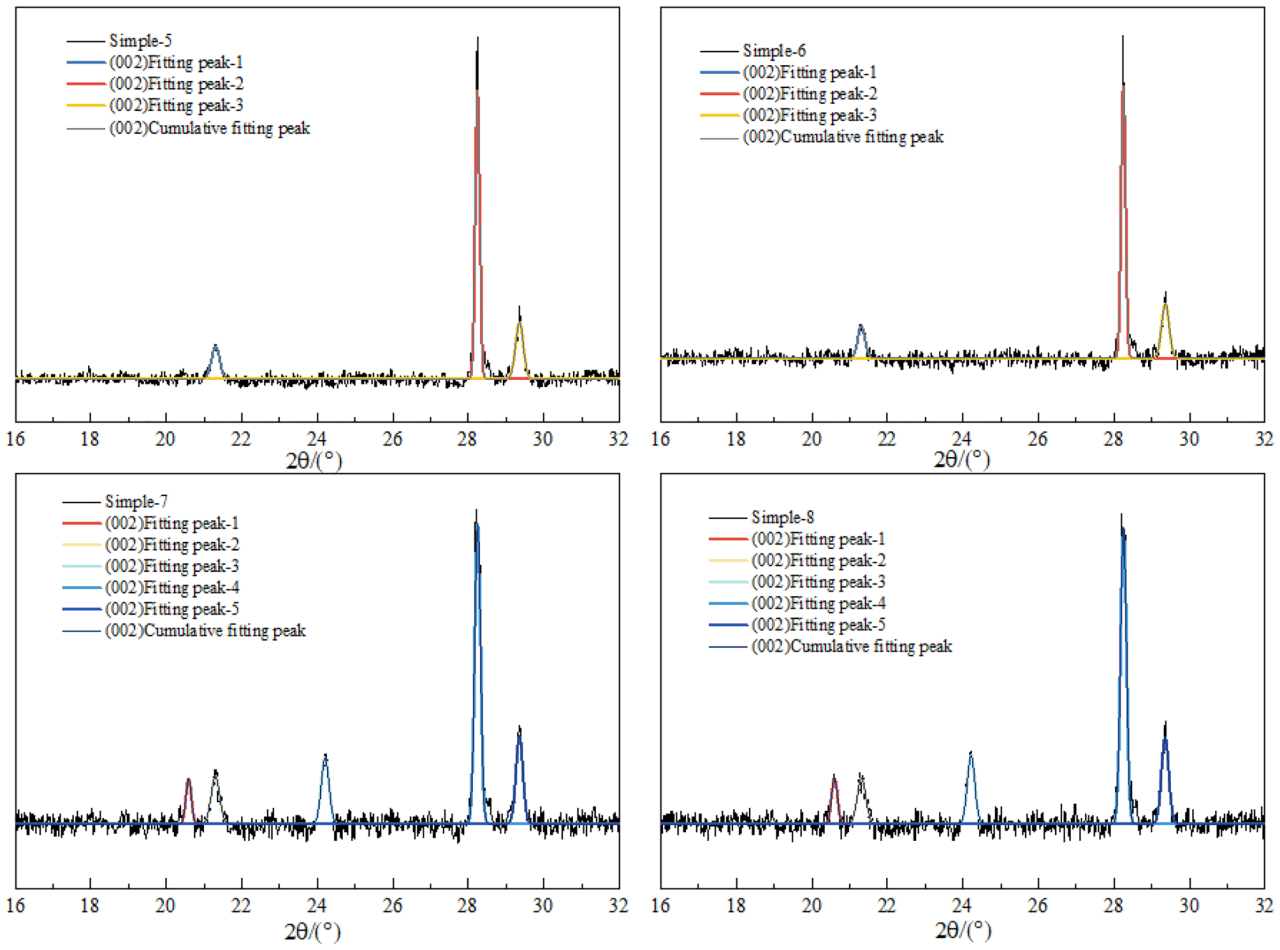
XRD curve fitting results for 002 peak of coal samples (5–8).

The composition of the raw coal did not undergo significant changes before and after the coal dust detonation. This is because the fixed carbon in the raw coal and the volatile matter in the coal powder pores are the main fuel for the detonation. To further study the decoupling of coal powder crystals during the detonation process, the method of analyzing the XRD (002) peak is proposed. There is a characteristic (002) peak near 2θ = 26°, The orientation degree of the aromatic ring carbon network layers can be determined based on the (002) peak, with its position reflecting the stacking distance of the aromatic ring layers. The peak fitting result of the (002) peak in the XRD pattern of the coal sample in the 2θ = 16–32° range is shown in Figs. 9 and 10. The microcrystalline structural parameters of the coal sample can be calculated based on the Bragges and Scherrer formulas from the XRD pattern. The structural parameters of coal, including intercrystalline spacing ${d}_{002}$, microcrystalline stacking height ${L}_{c}$., coal aromaticity ${f}_{a-XRD}$ and average layer number ${N}_{ave}$ of crystal stacking, can be obtained by the following formula^39^

1 $$d_{{002}} = \frac{\lambda }{{2{\text{sin}}\uptheta _{{002}} }}$$

2 $$L_{{\text{c}}} = \frac{{{\text{0}}{\text{.89}}\lambda }}{{B_{{002}} {\text{cos}}\uptheta _{{002}} }}$$

3 $$f_{{{\text{a - XRD}}}} = \frac{{A_{{002}} }}{{A_{{002}} + A_{\uplambda } }}$$

4 $$N_{{{\text{ave}}}} = \frac{{L_{{\text{C}}} }}{{d_{{002}} }} + 1$$

where, λ is the wavelength of X-rays, λ = 0.154 mm;*θ*_002_ is the central position of the (002) peak; ${\text{B}}_{002}$ is the full width at half maximum of the 002 peak; ${\text{A}}_{002}$ and *A*_λ_ are the peak areas of the (002) peak and γ peak, respectively.

As shown in Table 2, d002 can aracterize the distance between adjacent aromatic layer sheets in the internal structure of coal powder. The crystal spacing d values of coal powder sample 1 and sample 2 in the 8305# coal mining face area are 0.3517 nm and 0.3495 nm respectively, while the crystal spacing d values of coal powder sample 3 and sample 4 after detonation are 0.3436 nm and 0.3432 nm; Outside the coal mining face, the interplanar spacing (d_002_) of coal dust samples 5 and 6 is 0.3554 nm and 0.3571 nm, respectively. After the detonation, the interplanar spacing (d_002_) of samples 7 and 8 is 0.3412 nm and 0.3435 nm, respectively, with a small range of change before and after the detonation. L_c_ can be used to characterize the degree of coal structure ordering. The L_c_ of coal samples 1 and 2 from the 8305# coal mining face are 1.834 nm and 1.651 nm, respectively, while the Lc of samples 3 and 4 after detonation are 0.975 nm and 0.887 nm; The L_c_ of coal samples 5 and 6 outside the coal mining face are 1.323 nm and 1.248 nm, respectively, while the L_c_ of coal samples 7 and 8 after the detonation are 0.654 nm and 0.638 nm. It can be observed that the coal powder L_c_ on the external working face is lower than that of the working face coal powder. This may be due to the fact that the external coal has been exposed to air for a longer period of time, leading to a higher degree of oxidation, resulting in an increase in side chains and heteroatomic functional groups. At the same time, the coal powder undergoes detonation, reducing the stacking degree of the coal's aromatic layer.The f_a-XRD_ of coal samples 1 and 2 on the working face are 0.571 and 0.612 respectively, while the f_a-XRD_ of coal sample 3 and 4 after the detonation are 0.684 and 0.713. The f_a-XRD_ of coal samples 5 and 6 outside the working face are 0.553 and 0.492 respectively, and after the detonation, the f_a-XRD_ of coal samples 7 and 8 are 0.748 and 0.815. The detonation effect increases the aromaticity of the coal powder. The degree of stacking of microcrystalline structure units can be characterized by the average number of crystal stacking layers N_ave_. The N_ave_ of coal samples 1 and 2 inside the mining working face are 5.125 and 5.657 respectively; the values of coal samples 3 and 4 after detonation are 4.951 and 5.024 respectively. The N_ave_ of coal samples 5 and 6 outside the coal mining face are 5.366 and 5.412 respectively, while the N_ave_ value of coal samples 7 and 8 after the detonation are 4.859 and 4.946 respectively. Due to the greater aromaticity of coal powder, its detonation reactivity is weaker. Therefore, based on the chemical structure and microcrystalline characteristics of coal itself, it can be inferred that the explosiveness of coal dust in the mining face is greater than that of the coal sample outside the mining face, and at the same time greater than the coal dust sample after detonation.

#### FTIR analysis of solid products of pulverized coal detonation

**Figure 11:**
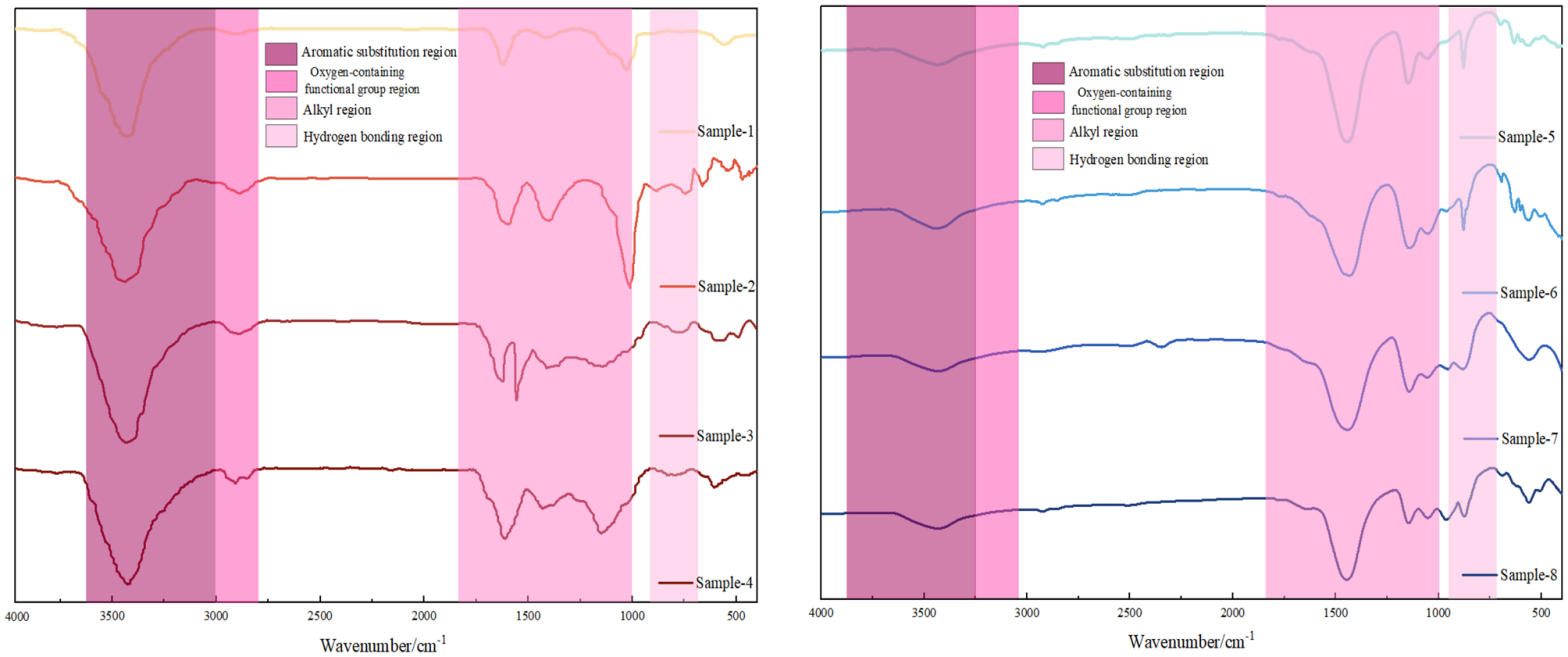
FTIR spectra of explosive and non-explosive products from different coal samples (1–8).

The FTIR spectrum of the coal sample can be divided into four regions based on the characteristics of absorption peaks: the hydrogen bond region (WB: 3650–3000 cm^−1^), the aliphatic region (WB: 3000–2800 cm^−1^), the oxygen-containing functional group region (WB: 1800–1000 cm^−1^), and the aromatic ring substitution region (WB: 900–700 cm^−1^). The FTIR spectrum of the coal sample after smoothing and baseline correction is shown in Fig. 11.

**Figure 12:**
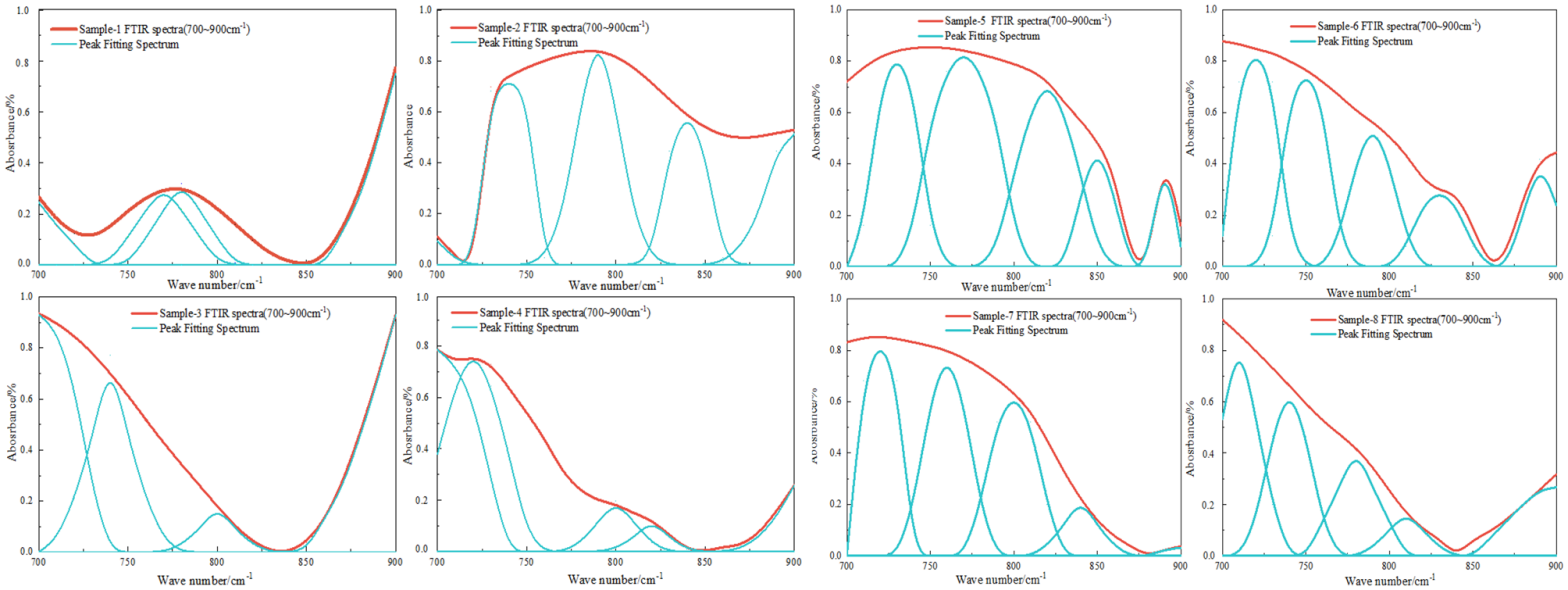
FTIR peak fitting of the aromatic structure infrared spectra of coal samples pre- and post-detonation.

The FTIR spectrum positions of the main functional groups before and after the coal sample detonation are shown in Table 3. The functional groups within four bands of the spectrum were analyzed using ORIGIN software for peak fitting, and the results are shown in Fig. 12.

**Table 3:** Band distribution of different functional groups in FTIR spectra.

No	Pos./cm^−1^	FG	No	Pos./cm^−1^	FG
1	740–750	Penta-substituted benzene	12	1685–1708	–COOH stretch vibration
2	760–790	Tetra-substituted benzene	13	2835–2850	–CH_2_ symmetric stretch vibration
3	800–835	Tri-substituted benzene	14	2858–2876	–CH_3_ symmetric stretch vibration
4	875–900	Di-substituted benzene	15	2895	–CH stretch vibration
5	1022–1040	C–O–C stretch vibration	16	2920	–CH_2_ asymmetric stretch vibration
6	1086–1098	Ether C–O vibration	17	2955	–CH_3_ asymmetric stretch vibration
7	1150–1274	Phenol hydroxyl C–O stretch vibration	18	3155–3240	Aromatic ring –OH
8	1365–1395	–CH_3_ symmetric bend vibration	19	3260–3320	OH–O
9	1432–1446	–CH_2_ anti-symmetric bend vibration	20	3420	OH–OH
10	1586–1619	Aromatic hydrocarbons C=C stretch vibration	21	3530	OH–π
11	1632–1657	Conjugate C=O	22	3590–3640	Liminal state –OH

**Figure 13:**
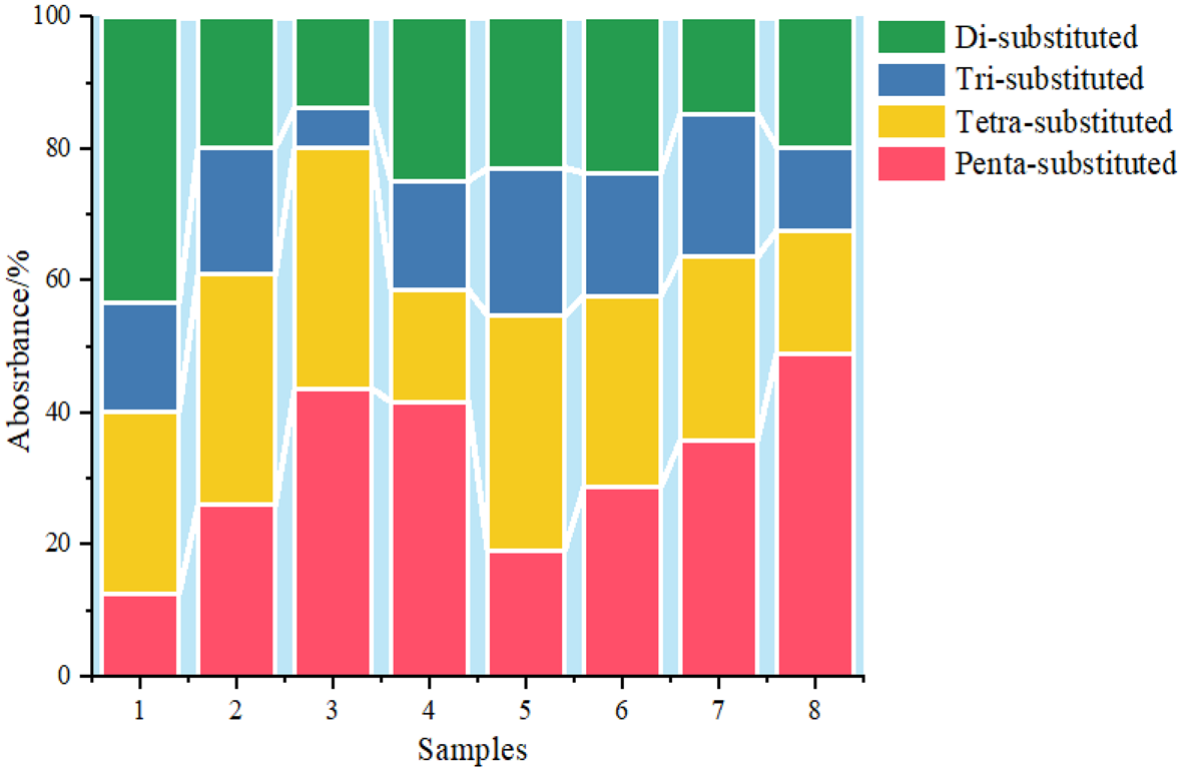
Benzene ring percentage of different coal samples pre- and post-detonation.

As shown in Fig. 13, the Penta-substituted benzene proportions of coal samples 1, 2, 3 and 4 are 12.65%, 26.12%, 43.64% and 41.68%, respectively. To aid comparison, it is evident from the percentage of benzene rings in various coal samples in Fig. 13: due to the impact of detonation burning, the proportion of benzene ring di-substituted content in the post-detonation coal sample is significantly higher than that in the non-detonation coal sample; the proportions of benzene ring tri-substituted, benzene ring tetra-substituted, and benzene ring penta-substituted content in the post-detonation coal sample are slightly lower than those in the non-detonation coal sample.

The detonation and detonation of coal dust is an intricate chemical reaction sequence encompassing several phases: the coal dust heating, coal particle pyrolysis, volatile matter emission, volatile component ignition and burn, and the burn of the remaining solid carbon. In this sequence, coal's organic elements are chemically transformed into a variety of byproducts. Aromatic derivatives such as dimethylbenzene (for disubstituted) and trimethylbenzene (for trisubstituted), as well as tetra- and pentasubstituted compounds like tetramethylbenzene and pentamethylbenzene, can form during coal's pyrolysis and detonation. The creation and depletion of these compounds are influenced by the detonation conditions' temperature, pressure, oxygen levels, and the characteristics of the coal dust.Upon exposure to high temperatures, coal dust particles break down thermally, emitting volatiles. These volatiles' constituents undergo selective breakdown or conversion, influenced by their inherent chemical stability and reactivity. Disubstituted benzene compounds may experience less breakdown under detonation due to their enhanced stability. Conversely, compounds with tri-, tetra-, or pentasubstitution might be more susceptible to additional reactions or breakdown at elevated temperatures, given their increased number of substituents.

**Figure 14:**
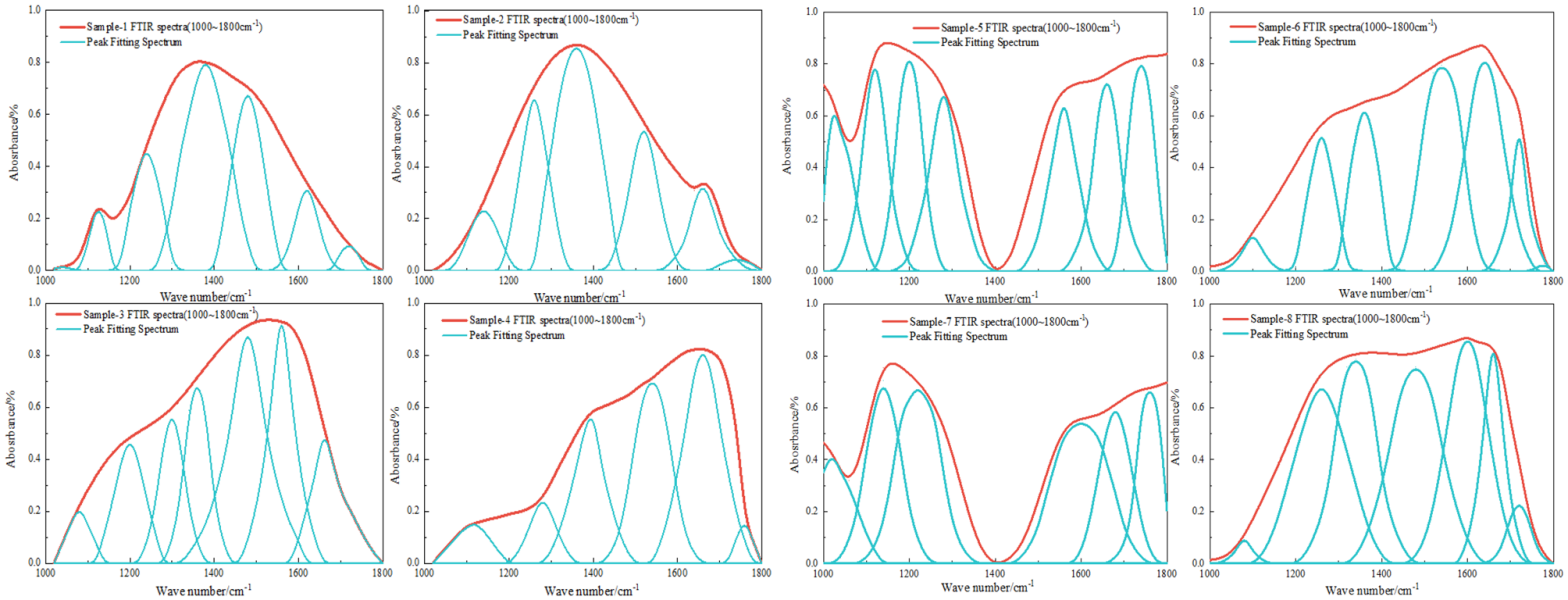
FTIR peak fitting of oxygen-containing functional groups in coal samples pre- and post-detonation.

Figure 14 shows the peak fitting results of the oxygen-containing functional groups in different coal samples before and after detonation. In coal samples 1 to 4, the phenolic hydroxyl C-O stretching vibration peaks are measured at 12.18%, 19.75%, 22.68%, and 19.52%, respectively. For samples 5 to 8, these proportions are 9.25%, 1.24%, 11.85%, and 1.68%. The heightened ether C–O vibration peak suggests an increase in oxygen-containing functional groups following the explosive detonation of coal dust. This is likely due to the high-temperature oxidation of hydrocarbons within the dust, resulting in a higher formation of oxygen-rich groups like ethers and phenols, a reaction that is facilitated by the intense heat of explosive detonation. In coal samples 1 through 4, the proportions of the –CH_3_ symmetric bending vibration peaks are 27.34%, 29.46%, 14.68%, and 20.72% respectively. For samples 5 through 8, these proportions are 1.05%, 26.97%, 0.68%, and 0.29%. The observed rise in the conjugated C=O peak suggests an increase in compounds with conjugated ketones or carboxylic acids during detonation combustion, likely due to oxidation or transformation of volatile compounds under such conditions, enhancing the presence of conjugated C=O groups. The conjugate C=O peaks in samples 1 to 4 show proportions of 17.54%, 19.86%, 20.21%, and 36.57%, and in samples 5 to 8, they are 22.98%, 29.18%, 27.97%, and 31.57%. An increase in these peaks implies more conjugated C=O group compounds, such as ketones or carboxylic acids, are formed during detonation combustion, attributed to the transformation of volatile compounds, leading to a rise in conjugated C=O group content.

**Figure 15:**
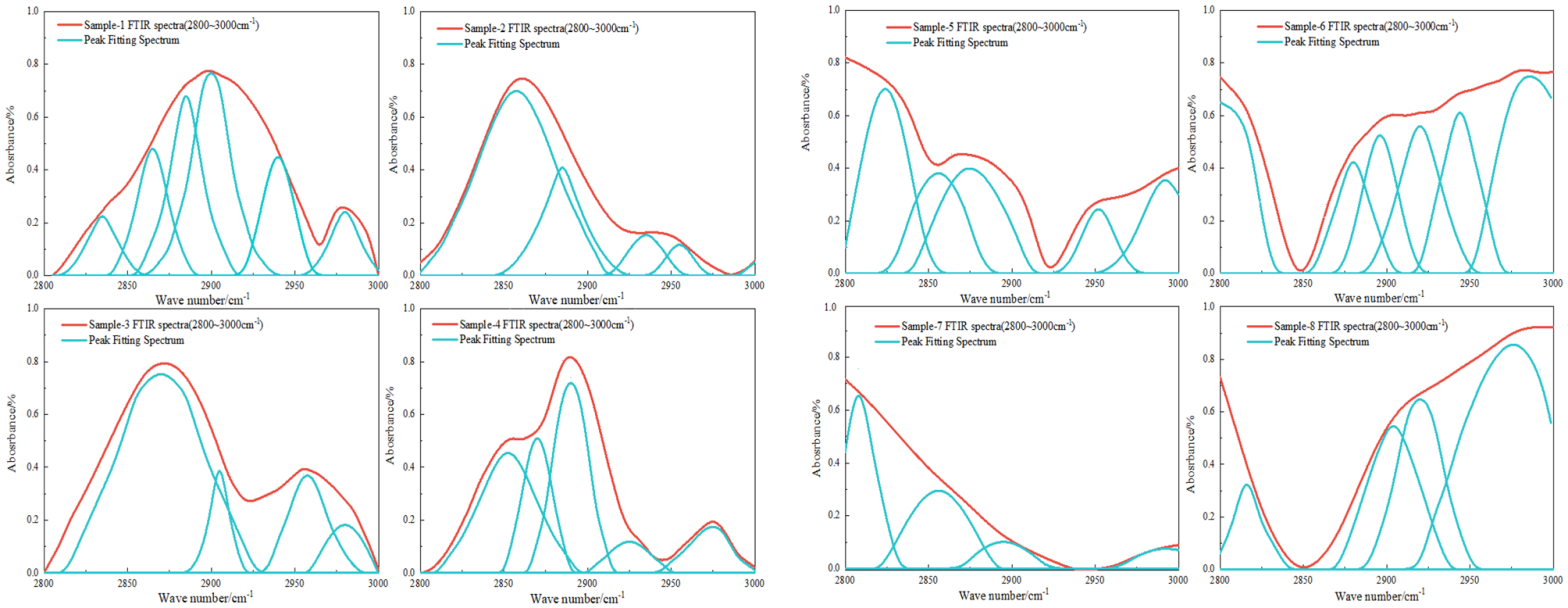
FTIR peak fitting chart of aliphatic structures in coal samples pre- and post-detonation.

Figure 15 shows the peak fitting results of aliphatic regions in coal samples.In coal samples 1 through 4, the –CH_2_ symmetric stretch vibration peak content percentages are 10.69%, 0.59%, 0.12%, and 0.09%, respectively. In contrast, samples 5 to 8 show proportions of 25.42%, 15.62%, 18.37%, and 8.26%. The pulverized coal's long-chain alkane structures are disrupted under extreme detonation conditions of high temperature and pressure, transforming the CH_2_ groups into different chemical configurations. This alteration could result in chain fragmentation into smaller entities or cyclization, creating aromatic compounds. Regarding the –CH_3_ symmetric stretch vibration peak content, samples 1 to 4 display 16.18%, 17.97%, 20.34%, and 26.45%, while samples 5 to 8 have 27.87%, 19.97%, 22.69%, and 29.25%. The increase in –CH_3_ groups at the reaction's end is due to chain scission or the formation of additional –CH_3_ terminal groups during pyrolysis or cracking.

**Figure 16:**
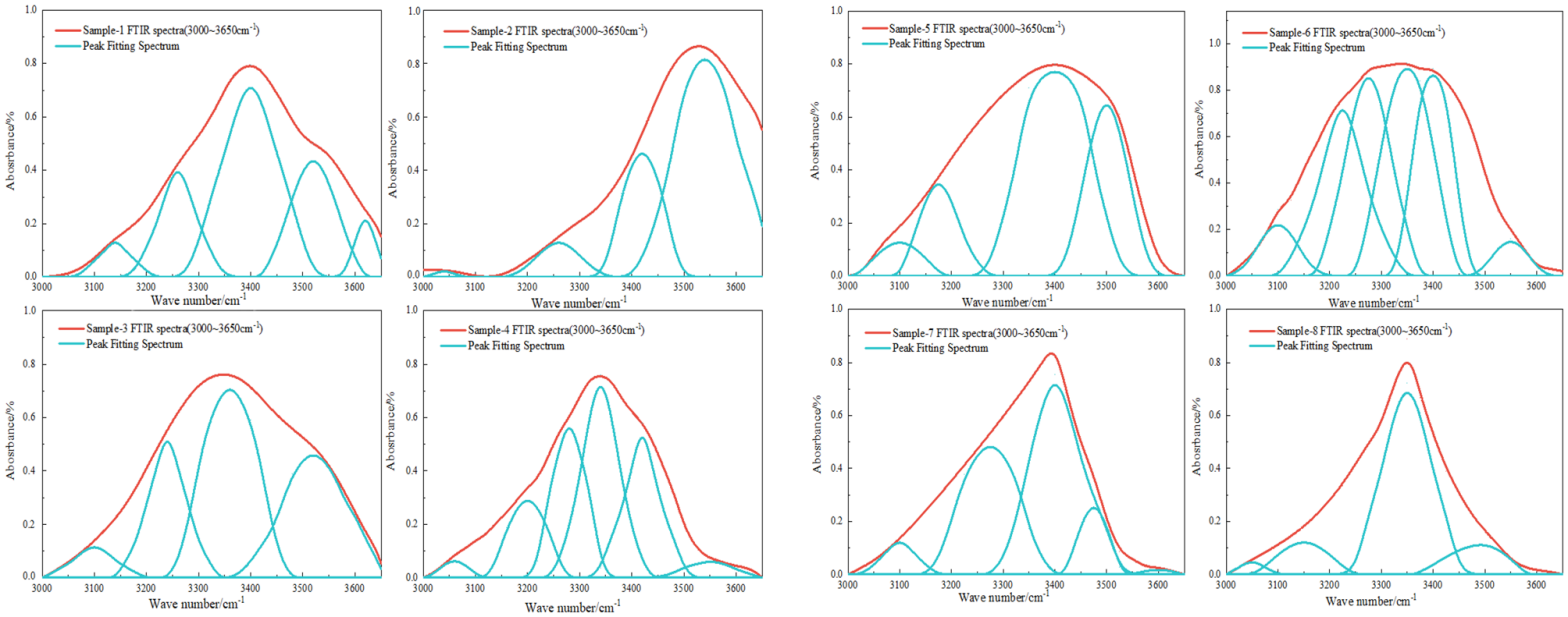
FTIR peak fitting diagram of various coal samples pre- and post-detonation.

Figure 16 shows the peak-to-peak fitting results of hydrogen bond region in coal samples. For the aromatic ring -OH peak content, samples 1 to 4 show 8.68%, 1.68%, 10.65%, and 4.54%, respectively, with samples 5 to 8 at 10.29%, 11.41%, 9.35%, and 11.97%. The OH-O peak content in samples 1 to 4 is 19.68%, 13.75%, 25.62%, and 25.86%, and in samples 5 to 8, it is 0.68%, 32.42%, 35.97%, and 32.48%. The elevated levels of aromatic ring –OH and OH–O peaks likely signify an augmented aromatic structure in the pulverized coal. During detonation combustion, non-aromatic hydrocarbon structures may be converted through pyrolysis and condensation into more stable aromatic rings. Concurrently, the OH groups might engage in addition reactions with these aromatic rings, raising the aromatic ring –OH content. An uptick in OH–O peaks could suggest the formation of additional phenolic or ether compounds. These hydroxyl groups may partake in dehydration reactions or combine with other groups, forming water or other small molecular structures, thus reducing their characteristic peaks in FTIR spectra.

Using the peak fitting results of FTIR spectra and the following formula, the aromatic ring condensation degree (DOC), aromatic hydrocarbon chain length (CH_2_/CH_3_), coal aromaticity index(*I*), and the aromatic carbon content (*f*_*a*_) before and after detonation can be calculated for different coal samples.

The calculation formula of infrared aryl carbon ratio of coal sample is as follow^40^:

5 $$DOC = \frac{{A_{{700 \sim 900}} }}{{A_{{1600}} }}$$

6 $$f_{a} = 1 - \frac{{C_{{al}} }}{C}$$

7 $$\frac{{{\text{C}}_{{{\text{al}}}} }}{{\text{C}}} = \left( {\frac{{{\text{H}}_{{{\text{al}}}} }}{{{\text{H}}_{{{\text{al}}}} {\text{ + H}}_{{{\text{ar}}}} }}{ + }\frac{{\text{H}}}{{\text{C}}}} \right){/}\frac{{{\text{H}}_{{{\text{al}}}} }}{{{\text{C}}_{{{\text{al}}}} }}$$

8 $$\frac{{{\text{H}}_{{{\text{al}}}} }}{{{\text{H}}_{{{\text{al}}}} {\text{ + H}}_{{{\text{ar}}}} }} = \frac{{{\text{A}}_{2800 \sim 3000} }}{{{\text{A}}_{2800 \sim 3000} {\text{ + A}}_{700 \sim 900} }}$$

where, *C*_*al*_ denotes the fatty carbon content in coal (%); *C* denotes the total amount of carbon (%); *H*_*al*_ denotes the content of fatty hydrogen (%); *H* denotes the total hydrogen content (%); *H*_*ar*_ denotes the relative content of aromatic hydrogen(%); *C*_*al*_ denotes the carbongen content in aliphatic hydrocarbon (%); *H*_*al*_/*C*_*al*_ is the usual experience value 1.8; *A*_*2800*~*3000*_ and *A*_*700*~*900*_ are the peak areas of aliphatic region and aromatic ring substitution region in FTIR spectral fitting results, respectively.

The aromatic ring condensation degree of coal can be represented by the ratio of the intensity of aromatic C–C vibration near 1600 cm^−1^ to the aromatic ring substitution area, as shown in Eq. (9):

9 $$DOC = \frac{{A_{{700\sim 900}} }}{{A_{{1600}} }}$$

The CH_2_/CH_3_ ratio can indicate the aromatic hydrocarbon chain length of the coal sample, with a higher value indicating a longer aliphatic chain in the coal. The calculation formula is as shown in Eq. (10):

10 $$\frac{{{\text{CH}}_{2} }}{{{\text{CH}}_{3} }} = \frac{{{\text{A}}({\text{CH}}_{2} )}}{{{\text{A}}({\text{CH}}_{3} )}} = \frac{{A_{{2835\sim 2850}} }}{{A_{{2858\sim 2876}} }}$$

The *I* can represent the abundance of aromatic hydrocarbons relative to aliphatic hydrocarbons in coal. The calculation formula is shown in Eq. (11):

11 $$I = \frac{{A_{{700\sim 900}} }}{{A_{{2800\sim 3000}} }}$$

According to the FTIR peak fitting of 8 coal samples, the peak areas of major functional groups were obtained, and the specific results were shown in Table 4.

**Table 4:** Peak area distribution of major functional groups in 8 coal samples.

Sample	*A*_700~900_	*A*(C=C)_700~900_	*A*(C=O)_1632~1657_	*A*(CH_2_)_2920_	*A*(CH_3_)_2955_	*A*_2800~3000_
1	34.64	513.69	110.85	27.48	12.97	95.82
2	65.43	846.17	182.57	64.44	10.37	141.18
3	83.54	418.64	97.73	54.68	48.68	169.08
4	110.16	812.36	81.01	44.57	42.39	194.59
5	88.91	647.1	73.82	18.06	10.96	20.67
6	86.64	660.32	94.64	12.84	7.35	19.11
7	120.71	598.63	51.47	1.58	1.27	3.14
8	125.98	657.07	47.38	1.32	1.01	2.98

Table 5 presents the data from Table 3 alongside the outcomes of formulas (5)–(11). Examination of Table 4 reveals that the Degree of Aromatic Condensation (*DOC*) for coal samples 3 and 4 is 2.96 and 1.75 times higher than that of samples 1 and 2, respectively. Similarly, samples 7 and 8 show DOC values 1.25 and 1.13 times greater than samples 5 and 6. The Index of Aromaticity (*I*) for samples 3 and 4 is 1.36 and 1.22 times the I values of samples 1 and 2, while for samples 7 and 8, it is 7.61 and 7.17 times the I values of samples 5 and 6. This suggests a positive correlation between the degree of aromatic condensation (*DOC*) and the index of aromaticity (*I*) with the intensity of detonation. The correlation may stem from high-temperature conditions causing the breakdown of non-aromatic structures in coal, such as alkyl side chains, leading to the release of gases like methane. This increases the proportion of aromatic ring structures, which could further condense into polycyclic aromatic hydrocarbons or larger aromatic entities, thus elevating the *DOC* and *I* values. Post-detonation combustion, the aromatic carbon content in coal appears to rise, enhancing its aromaticity. Conversely, the CH_2_/CH_3_ ratios for coal samples 3 and 4 are only 0.53 and 0.17 times those of samples 1 and 2. For samples 7 and 8, these ratios are 0.75 and 0.74 times those of samples 5 and 6, indicating a negative correlation between aromatic chain length and detonation combustion. High temperatures during detonation likely cause longer aromatic chains to break down into shorter structures, thereby decreasing the average aromatic chain length. At the same time, the rapid energy release of detonation combustion may also promote the rearrangement of aromatic structures in coal to form a more stable polycyclic aromatic structure, and may further promote the condensation of aromatic rings through dehydrogenation and free radical reaction.

**Table 5:** FTIR structural parameters of 8 coal samples.

Sample	DOC	CH_2_/CH_3_	*I*	*f*_a_
1	0.0674	2.1187	0.3615	0.2938
2	0.0773	6.2141	0.4635	0.2809
3	0.1996	1.1233	0.4941	0.3198
4	0.1356	1.0514	0.5661	0.2873
5	0.1374	1.6478	4.3014	0.0681
6	0.1312	1.7469	4.5338	0.0703
7	0.2016	1.2441	38.4427	0.0114
8	0.1917	1.3069	42.2752	0.0101

Moreover, the *f*_a_ values for coal samples 3 and 4 are 1.05 and 1.02 times those of samples 1 and 2, while for samples 7 and 8, they are 0.195 and 0.186 times those of samples 5 and 6. This significant variance in the *f*_a_ ratio post-detonation combustion both within and beyond the mining face suggests a link to the oxidation levels in the two types of uncombusted raw coal. Further investigation is warranted to elucidate the underlying reasons.

## Preliminary exploration of RDW transmission mechanism

The formation and transmission mechanism of rotating detonation wave (RDW) are essentially the same. For rotating detonation chamber (RDC), it is crucial to reveal the propagation characteristics of RDW under different coal powder conditions for its stable operation, flexible control, and future engineering applications. The structure of RDW is transient and three-dimensional, but the classic detonation wave ZND two-dimensional theoretical model can exhibit an average structure in the cross-section perpendicular to the direction of propagation.

**Figure 17:**
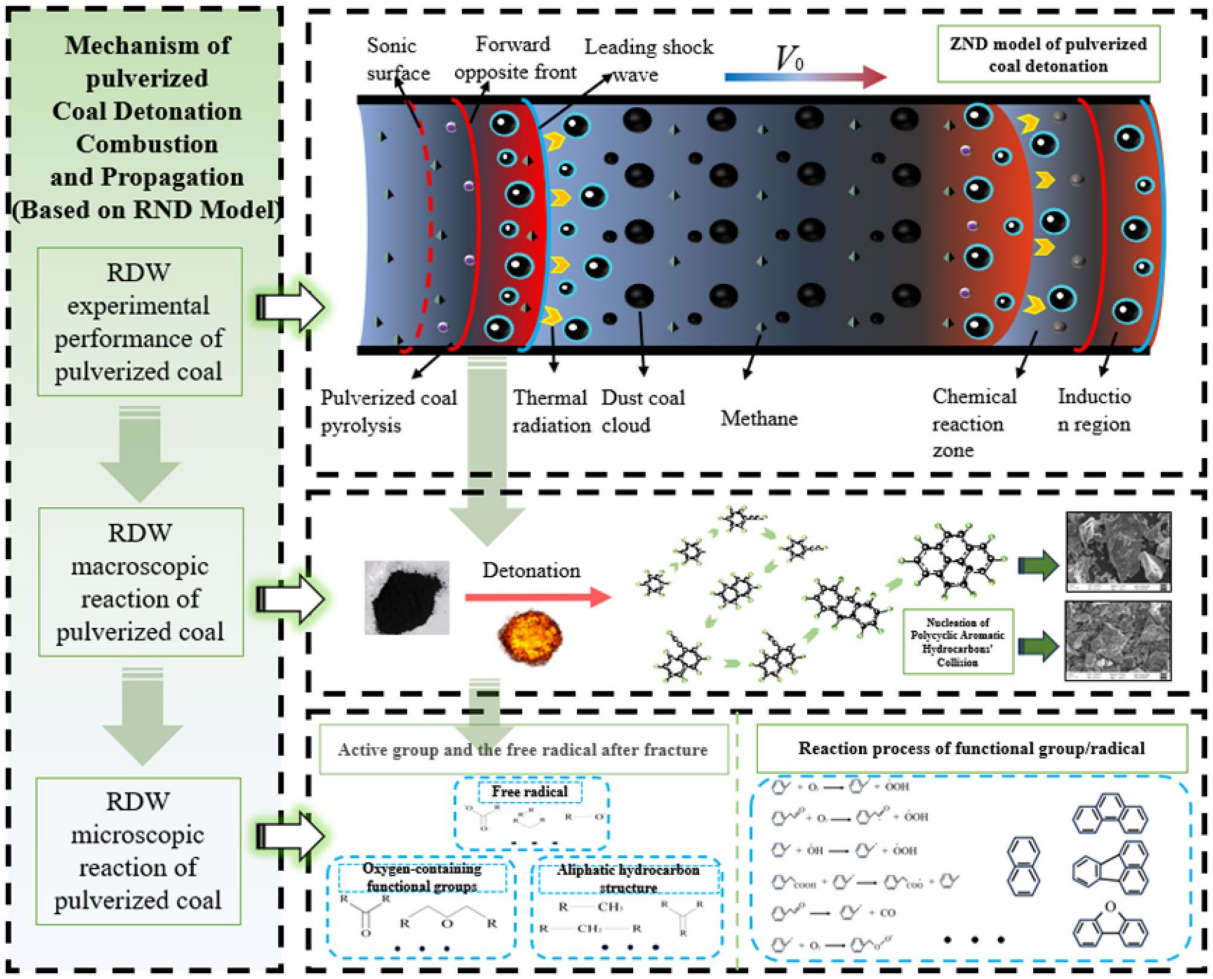
Mechanism of coal powder RDW propagation based on ZND model.

The structure of the coal detonation wave ZND model is illustrated in Fig. 17, the unburned coal dust moves to the right with velocity (*V*_0_). In the coal/methane/oxygen mixed detonation, the leading shock wave heats the coal particles, creating a flammable mixture layer in the devolatilization zone that envelops each coal particle. Within the reaction zone, coal particles, the volatiles of coal particles, and CH_4_ methane coexist. The shock wave compresses and heats the reactants, follow this study by an induction zone, where molecules heated by the shock wave undergo thermal decomposition to produce active groups. It is widely accepted that although the mixture in the induction zone is heated by the shock wave, its thermodynamic state remains fundamentally unchanged. This is succeeded by the chemical reaction zone, where the rapid release of chemical energy results in further temperature increase, and pressure and density decrease. At high initial pressures, with a higher oxygen content, more fuel can be oxidized, leading to faster and stronger detonation reactions.

After passing through the leading shock wave, it heats up in the induced area without detonation reaction, continuously maintaining the propagation of RDW by releasing energy. The formation of coal dust detonation granular products mainly relies on the collision and nucleation of polycyclic aromatic hydrocarbons. It can be inferred that the addition and extraction of phenyl group H, as well as the precipitation of C_2_H_2_, play a dominant role in the evolutionary process. The oxygen-containing groups in the coal powder participate in oxidation, pyrolysis, and condensation reactions under strong shock wave conditions, releasing heat that diffuses to excite the unreacted coal powder, causing more chemical bond breakage and the generation of more active groups. This leads to the accumulation of heat in a certain volume, further accelerating the reaction process, resulting in high temperature and high-velocity RDW. These findings not only deepen our understanding of the mechanism of coal dust detonation, but also provide important theoretical basis for the future control and application of coal dust detonation.

## Conclusions

In this study, the system investigated the characteristics of coal dust rotation detonation at different positions of underground coal mine working faces. A detailed comparison of the degree of material changes before and after the detonation was conducted, leading to a preliminary understanding of the detonation mechanism of coal dust during detonation. The following can be concluded:

- Under the same experimental conditions, the coal dust inside the underground coal mining face exhibits a stronger detonation characteristic (detonation success rate, high-velocity RDW, economy, particle size advantage, and persistence) compared to the coal dust outside the mining face. This pattern of change can offer guidance for the application of explosion technology, especially in the advancement and processes of coal mining machines based on new explosion coal mining techniques.
- Through XRD studies of the products before and after detonation, it was found that detonation increases the aromaticity of coal powder while reducing the stacking degree of the coal's aromatic layer.
- Through FTIR qualitative and quantitative analysis, it was found that the aromatic condensation degree (*DOC*) and aromaticity index (*I*) are positively correlated with the detonation combustion intensity of coal, indicating that high temperature conditions enhance the aromatic structure of coal. Additionally, the study found that the aromatic chain length is negatively correlated with detonation combustion, indicating that high temperature decomposes longer aromatic chains into shorter ones, affecting the structural composition of coal.
- Research on the products of coal dust detonation indicates that the formation of particle products is significantly influenced by the collision and nucleation of polycyclic aromatic hydrocarbons, in which the addition and extraction of phenyl H, as well as the precipitation of C_2_H_2_, play a crucial role. Under strong shock wave conditions, oxygen-containing groups in coal powder participate in oxidation, pyrolysis, and condensation reactions, releasing heat to accelerate the reaction process, resulting in the generation of high-temperature and high-velocity rotating detonation waves (RDW).

In conclusion, this study not only addresses the critical scientific and technical challenges associated with the application of pulverized coal in RDW technologies but also provides a clear pathway for future development in this domain. By elucidating the detonation characteristics of coal dust and its implications for in-situ coal mining, our research plays a pivotal role in advancing the efficiency, safety, and sustainability of coal energy utilization and mining practices.

## Data Availability

The datasets used and/or analysed during the current study available from the corresponding author on reasonable request.
